# Efficacy of a formulation of sarolaner/moxidectin/pyrantel (Simparica Trio^®^) for the prevention of *Thelazia callipaeda* canine eyeworm infection

**DOI:** 10.1186/s13071-022-05501-6

**Published:** 2022-10-16

**Authors:** Marcos Antonio Bezerra-Santos, Jairo Alfonso Mendoza-Roldan, Giovanni Sgroi, Riccardo Paolo Lia, Giulia Venegoni, Fabrizio Solari Basano, Roose Nele, Sean P. Mahabir, Stasia Borowski, Thomas Geurden, Domenico Otranto

**Affiliations:** 1 Department of Veterinary Medicine, University of Bari, Valenzano, Bari, Italy; 2 Arcoblu s.r.l, Milan, Italy; 3 Zoetis, Mercuriusstraat 20, 1930 Zaventem, Belgium; 4 Zoetis, Portage Street 333, Kalamazoo, MI 49007 USA; 5 Department of Pathobiology, Faculty of Veterinary Science, Bu-Ali Sina University, Hamedan, Iran

**Keywords:** *Thelazia callipaeda*, Prevention, Dogs, Moxidectin, Simparica Trio, Zoonosis

## Abstract

**Background:**

For a long time known as the oriental eyeworm, *Thelazia callipaeda* is a zoonotic nematode that infects the eyes of a wide range of vertebrate hosts including dogs, cats, wildlife carnivores, lagomorphs, and humans. The high occurrence of this infection in Europe and the first cases in the United States have increased scientific interest in the parasite, as it also represents a risk for people living in endemic areas. Therefore, treatment and prevention of thelaziosis in canine population are advocated to reduce the risk of human infection as well. Here, we assessed the efficacy of a formulation containing sarolaner/moxidectin/pyrantel (Simparica Trio^®^) administered orally at monthly intervals, for the prevention of establishment of infection with *T. callipaeda* in naturally infected dogs. In this formulation, moxidectin is expected to have efficacy against eyeworms, whereas sarolaner and pyrantel are not.

**Methods:**

The study was conducted in eyeworm endemic areas of Italy and France, where dogs (*n* = 125) were assigned into two groups consisting of a negative control group (G1; *n* = 62), in which animals were treated monthly with a control product (sarolaner; Simparica^®^), and a treatment group (G2; *n* = 63) in which animals were treated monthly with Simparica Trio (sarolaner/moxidectin/pyrantel) from day 0 to day 150. In total, nine animals were withdrawn from the study (two animals became positive at day 30, and seven for reasons unrelated to eyeworm infection), resulting in 116 animals (*n* = 58 for G1; *n* = 58 for G2).

**Results:**

In G1, 16 out of 58 animals (27.6%) were observed with eyeworms during the study, and none of the animals from G2 were ever observed with eyeworms, resulting in 100% efficacy (*P* < 0.0001) in the prevention of establishment of *T. callipaeda* infection. Adult nematodes and fourth-instar (L4)-stage larvae were recovered from the eyes of positive animals, counted, and morphologically identified as *T. callipaeda*. In addition, specimens from Italy were molecularly confirmed as belonging to the haplotype 1 (i.e., the only one circulating in Europe so far).

**Conclusions:**

Data presented herein demonstrated 100% efficacy of Simparica Trio for the prevention of *T. callipaeda* eyeworm infection in dogs from highly endemic areas of France and Italy. The use of this formulation is advantageous, as it is a licensed product in Europe with a wide efficacy spectrum against other nematodes, multiple tick species, and fleas. In addition, preventing the development of infection in dogs could also be a prophylaxis measure for zoonotic *T. callipaeda* infection in humans inhabiting endemic areas.

**Graphical Abstract:**

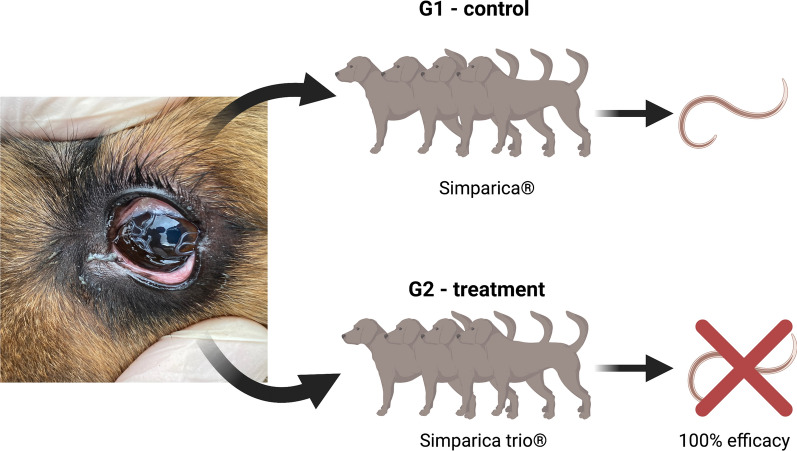

**Supplementary Information:**

The online version contains supplementary material available at 10.1186/s13071-022-05501-6.

## Background

*Thelazia callipaeda* is a zoonotic nematode widely distributed in Asia and Europe [1]. This parasite, once known as the oriental eyeworm, infects a wide range of vertebrate hosts including domestic animals (e.g., dogs and cats), wildlife fauna (e.g., beech martens, bears, foxes, jackals, wolves), and humans [2, 3], causing ocular infections characterized by different degrees of conjunctivitis, follicular hypertrophy of the conjunctiva, discomfort, epiphora, itchiness, congestion, swelling, hypersensitivity to light, and keratitis [4]. The vectors of this nematode are drosophilid fruit flies of the species *Phortica variegata* in Europe, and *Phortica okadai* in Asia [5]. However, a third species, *Phortica oldenbergi*, has been demonstrated as a potential vector of *T. callipaeda* under experimental conditions [6]. In Europe, since the first report of this nematode in Italy [7], several other cases have been reported in other countries in different domestic and wild animal species, indicating a wide vertebrate host range for *T. callipaeda*, and supporting wild carnivores as important players on the spreading of this eyeworm in endemic areas, as well as in remote environments [1–3, 8].

The high prevalence of *T. callipaeda* in dogs from specific geographical areas in Italy (up to 41.76% [9]), Spain (up to 61.3% [4]), Serbia (up to 35.52% [10]), and France (no prevalence data, but a high number of reported cases [11]); suggest that stable endemic foci of the infection occur [12]. Conversely, lower prevalence in Switzerland (i.e., 5.3% [13]) and Portugal (i.e., 3.8% [14]) may indicate the emergence of this parasite in previously non-endemic countries [12]. The high occurrence of this infection in dogs is also risky for people living close to them, as the vector, *P. variegata*, feeds on lachrymal secretions of several vertebrate hosts, including dogs and humans [15]. Therefore, treatment and prevention of this infection in dogs from endemic areas is advocated to reduce the risk to human health as well.

Different macrocyclic lactone formulations have been tested against *T. callipaeda* infection in dogs (e.g., moxidectin 2.5% and imidacloprid 10%, milbemycin oxime/praziquantel, milbemycin oxime/afoxolaner) demonstrating efficacy ranging from 90 to 100% against this eyeworm infection [16–19]. In addition, the prevention of the eyeworm with either moxidectin and milbemycin oxime has been demonstrated in dogs from Italy, France, and Spain [19–21]. In order to widen the spectrum of preventive treatment options available for eyeworm infection and to provide a prophylaxis tool for new cases of infection in endemic areas, this study assessed the efficacy of Simparica Trio^®^ administered at the minimum doses of 1.2 mg/kg sarolaner, 24 µg/kg moxidectin, and 5 mg/kg pyrantel at monthly intervals, for the prevention of establishment of infection with *T. callipaeda* in dogs from highly endemic areas in Europe.

## Methods

### Study design

**Fig. 1:**
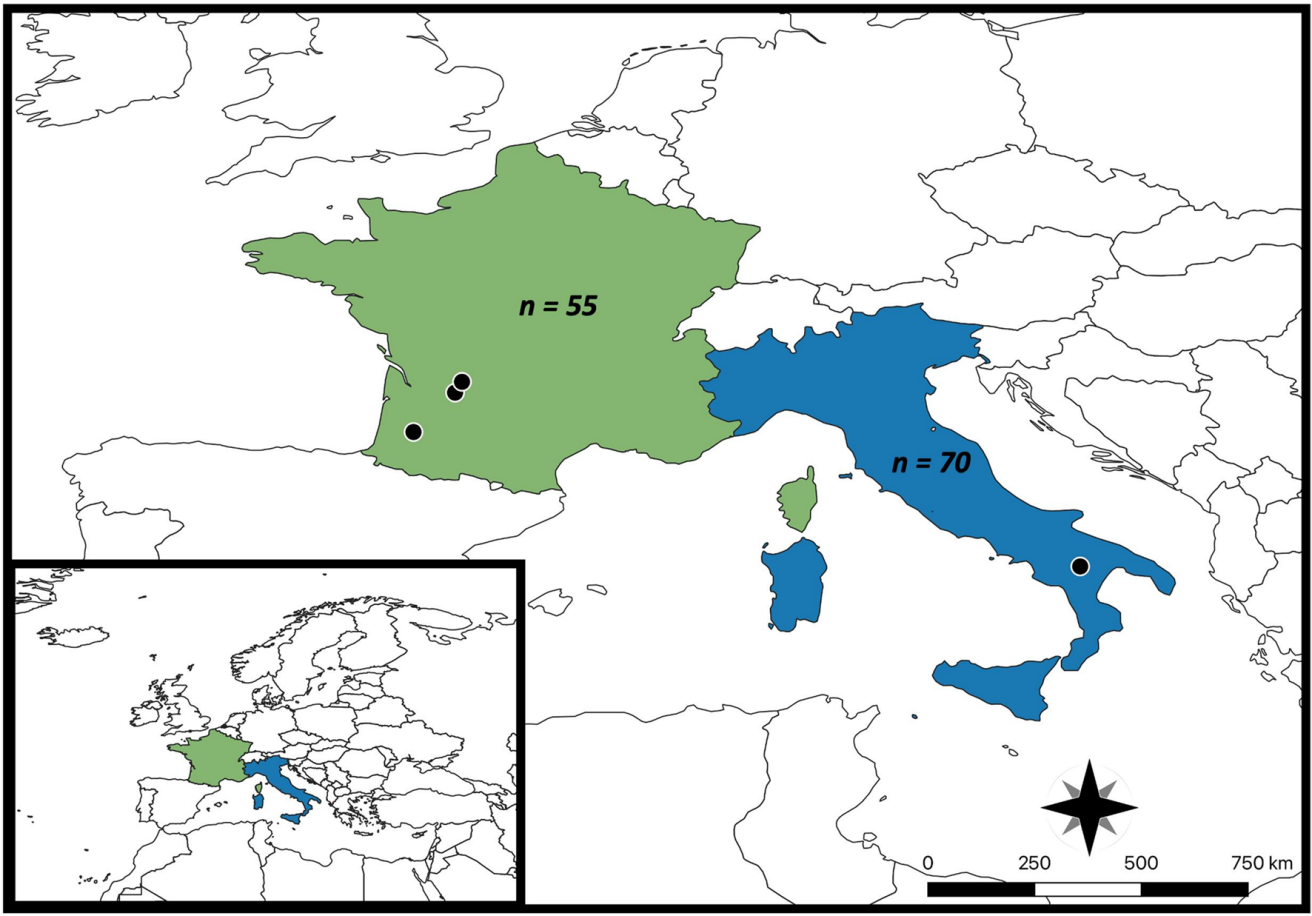
Map of the study area. Black dots show the exact geographical location in France and Italy where the study was performed

The study was conducted in the Basilicata region in southern Italy and in three veterinary clinics in the Nouvelle-Aquitaine region in France (Fig. 1). Both study areas are endemic for *T. callipaeda* eyeworms, with previously reported cases of thelaziosis in dogs [9, 11, 22], as well as high abundance of *P. variegata* vectors [23]. The study was conducted under the principles of Good Clinical Practice (GCP), as a controlled, blinded, and randomized multi-center field study. An informed consent form was signed by the dog owners before inclusion in the study.

**Fig. 2:**
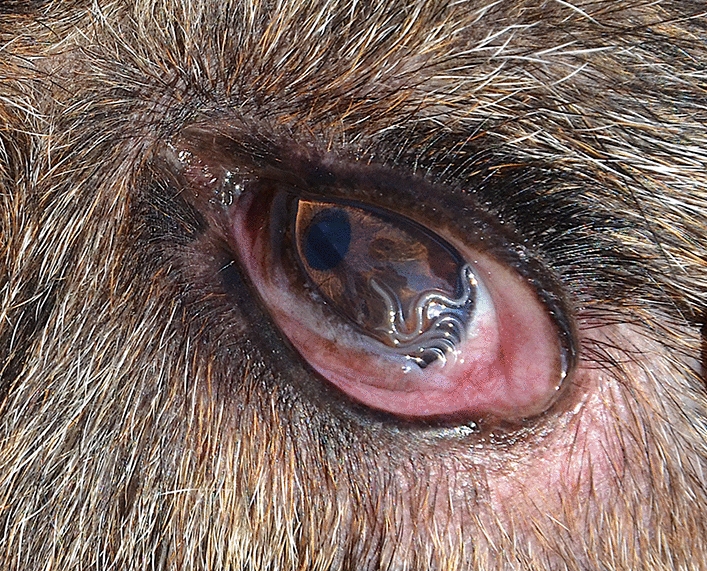
Eye of a dog positive for adult *Thelazia callipaeda* at the pre-screening period of the study

During a pre-screening visit (days − 28 to − 14), animals were clinically evaluated for suitability for inclusion in the study. All animals were examined for the presence of adult *T. callipaeda* worms in both eyes, including a thorough examination underneath the third eyelid and conjunctival pouch flushing with 5 ml of saline solution (0.9%), which was preserved in sterile tubes. After removal of *T. callipaeda* adult worms from the tubes, further centrifugation for 5 min at 700×*g* was performed, the supernatant was aspirated, and the sediment (1 ml solution) was analyzed under an optical microscope at ×40 magnification for the detection of nematode larvae. The collected nematodes (Fig. 2) were morphologically identified according to published keys [24, 25]. Following the pre-screening visit, all animals received a treatment with Milbemax^®^.

On day 0, animals were again physically evaluated, and ocular examination was performed in each dog. Animals that scored negative for *T. callipaeda* eyeworm and met all the criteria to be included in the study (i.e., ≥ 8 weeks of age, ≥ 2 kg, not pregnant or lactating, and not intended for breeding during the study) were enrolled and randomly assigned to treatment groups (G1: control product consisting of sarolaner [Simparica^®^]; and G2: investigated product consisting of sarolaner/moxidectin/pyrantel [Simparica Trio]), with both products presented as a flavored hard chewable tablet administrated orally. Follow-up treatments were provided on days 30 (± 3), 60 (± 3), 90 (± 3), 120 (± 3), and 150 (± 3) for all animals that remained negative for eyeworm infection, as diagnosed during ophthalmological assessment. If an animal was found positive for the presence of *T. callipaeda* adults on any of these follow-up treatment days, it was excluded from the study and received a commercial spot-on treatment with moxidectin/imidacloprid (Advocate^®^). All products were administered according to the European Union (EU) label instructions, and doses were selected according to the animal’s weight. At study completion on day 180 (± 3), animals received a general physical examination and ophthalmological assessment. Throughout the study period, all animals were observed daily by the owners for any health abnormality, and in the case of any adverse event, the investigator was contacted to examine the dog, evaluate whether the animal could continue in the study, and document diagnosis and concomitant treatment.

### Nematode identification

Nematodes collected from Italy and France were identified according to morphological keys [24, 25], and specimens from Italy were also identified by molecular characterization of partial (689 base pairs) mitochondrial cytochrome *c* oxidase subunit 1 (*cox*1) gene. Briefly, genomic DNA of worms collected from the eyes of positive dogs was extracted using the DNeasy Blood & Tissue Kit (Qiagen, Hilden, Germany). Conventional polymerase chain reaction (PCR) was performed using the primers NTF (5′-TGATTGGTGGTTTTGGTAA-3′) and NTR (5′-ATAAGTACGAGTATCAATATC-3′), and amplicons were purified and sequenced in both directions using BigDye Terminator v.3.1 chemistry in a 3130 Genetic Analyzer (Applied Biosystems, Waltham, MA, USA) in an automated sequencer (ABI Prism 377). Sequences were analyzed with MEGA7 software and compared with those available in the GenBank database through the BLAST search tool.

### Statistical analysis

A minimum sample size of 52 dogs per group was calculated based on the number of animals sufficient to detect a difference between infection rates for control product (G1) and treated (G2) animals with at least 80% power at the two-sided 5% significance level, assuming a maximum infection rate of 2% for treated animals and a minimum infection rate of 20% for control animals. A dog was considered positive as soon as an adult eyeworm was observed anytime during the study period. If adult eyeworms were observed on day 30, the dog was excluded from efficacy analyses, as the animal was assumed to have been infected before day 0. The proportion of eyeworm-free dogs was summarized in two-way frequency tables (eyeworm-free x treatment). The treatment was considered effective if the proportion of ever eyeworm-positive dogs in the treated group was significantly lower than in the control group and if it was at least 90% efficacious, using the following formula:

$$\%\, {\text{Efficacy}} = 100 \times \left( {\frac{{p_{{\text{C}}} - p_{{\text{T}}} }}{{p_{{\text{C}}} }}} \right),$$

where ${p}_{\mathrm{C}}$ is the proportion of animals infected for the control (G1) and ${p}_{\mathrm{T}}$ is the proportion of animals infected for the treated group (G2).

Fisher’s exact test was used to compare treatment groups for the presence of eyeworm during the study (yes/no). The test was conducted at the two-sided 0.05 significance level. Nematode counts were summarized by treatment group and stage (individual levels and overall) with descriptive statistics (mean, median, standard deviation, minimum and maximum).

## Results

A total of 125 dogs (*n* = 70 from Italy; *n* = 55 from France) were included in the study on day 0 (Table 1); however, nine animals were withdrawn during the study (two that became positive at day 30, and seven due to reasons unrelated to eyeworm infection), leaving 116 animals (*n* = 58 in G1; 58 in G2).

**Table 1:** Breed, sex, hair type, housing conditions and the number of dogs included in the study and receiving concomitant medication at enrolment

Characteristics	G1 (*n* = 62)		G2 (*n* = 63)	
	*n*	%	*n*	%
Breed				
Purebred	51	82.3	51	81.0
Non-purebred	11	17.7	12	19.0
Animal spends time				
Indoors and outdoors	19	30.6	20	31.7
Mostly outdoors	43	69.4	43	68.3
Sex				
Male	31	50.0	41	65.1
Female	31	50.0	22	34.9
Hair type				
Long	9	14.5	11	17.5
Medium	20	32.3	14	22.2
Short	33	53.2	38	60.3
Therapeutic/prophylactic medication				
On medication	2	3.2	4	6.3
Not on medication	60	96.8	59	93.7

G1: control group consisting of sarolaner (Simparica^®^). G2: investigated veterinary product consisting of sarolaner/moxidectin/pyrantel (Simparica Trio^®^)

*n*: number of dogs, %: percentage of all dogs

In the control group (G1), 16 out of 58 animals (27.6%) were observed with eyeworms during the study. In the treated animals (G2), no eyeworm infections were detected, indicating 100% efficacy (*P* < 0.0001) in the prevention of establishment of *T. callipaeda* infection in dogs for at least 28 days after treatment with Simparica Trio (Table 2).

**Table 2:** Frequency distributions for the presence of eyeworms after treatment in both groups of animals

Treatment group	Eyeworms				Total	
	Negative		Positive			
	*n*	%	*n*	%	*n*	%
G1 (Simparica)	42	72.4	16	27.6	58	100
G2 (Simparica Trio)	58	100	0	0	58	100

*n*: number of dogs, %: percentage of all dogs

Adult nematodes and L4-stage larvae were recovered from the eyes of positive animals, counted, and morphologically identified as *T. callipaeda* (Table 3), which were molecularly confirmed as belonging to haplotype 1 with 100% nucleotide identity with other *T. callipaeda* sequences (AM042549.1; OK662943.1) available in GenBank.

**Table 3:** Individual animal listing for nematode counts by development stage collected from the eyes of dogs belonging to G1

Treatment group	Animal	L1	L3	L4	L5	Adults	Total
G1 (Simparica)	1	–	–	–	–	3	3
	2	–	–	–	–	4	4
	3	–	–	–	–	2	2
	4	–	–	–	–	1	1
	5^a^	–	–	–	–	–	–
	6	–	–	–	–	4	4
	7	–	–	–	–	7	7
	8	–	–	–	–	2	2
	9	–	–	–	–	3	3
	10	–	–	2	–	1	3
	11	–	–	–	–	5	5
	12^a^	–	–	–	–	–	–
	13	–	–	3	–	–	3
	14	–	–	–	–	4	4
	15	–	–	–	–	2	2
	16	–	–	–	–	2	2

^a^Animals in which nematodes were observed but not recovered

Moderate or mild clinical signs were observed in *T. callipaeda*-positive animals from G1 at follow-up visits and consisted of conjunctivitis and ocular discharge (Additional file 1: Table S1).

## Discussion

The use of a monthly treatment with Simparica Trio resulted in 100% efficacy in preventing ocular thelaziosis by *T. callipaeda* in dogs from two different endemic areas in Europe, as such providing a reliable control strategy against this nematode in dogs. The prevention of thelaziosis in dogs using Simparica Trio was assessed through treatment followed by ocular examination every month over a 6-month period (May–November), which represents the period of the year when high numbers of infections are registered [9] due to the occurrence of *P. variegata* in the environment as well [5].

The high efficacy of Simparica Trio against *T. callipaeda* reported herein is due to the presence of moxidectin, which acts against diverse species of parasitic nematodes (e.g., *Toxocara canis*, *Ancylostoma caninum*) [26]. The combination with pyrantel complements the anthelmintic efficacy spectrum of action (against *Toxascaris leonina* and *Uncinaria stenocephala*), being also efficacious against *Dirofilaria immitis* [27] and *Angiostrongylus vasorum* [28]. In addition, the combination with sarolaner protects against infestation by ticks and fleas [29, 30]. Finally, the prophylactic efficacy of moxidectin, as well as that of milbemycin oxime (licensed product available), was found in previous studies to prevent infection by *T. callipaeda* [19–21], showing high efficacy of these two compounds.

Studies on the efficacy of diverse chemical compounds against *T. callipaeda* infection have been performed in dogs [16–20, 31] and cats [17, 32] using several formulations, including moxidectin 2.5% and imidacloprid 10% [18], milbemycin oxime/praziquantel [17], milbemycin oxime/afoxolaner [19], and fipronil/(S)-methoprene/eprinomectin/praziquantel [32]. Here, the oral formulation (minimal commercial dose of 1.2 mg/kg sarolaner, 24 µg/kg moxidectin, and 5 mg/kg pyrantel) has been proved 100% efficacious in preventing eyeworm infections in dogs, adding a new option available in the market for the prevention of *T. callipaeda* eyeworm infections. In addition, the wide spectrum of this oral formulation against other nematodes (i.e., *T. canis*, *A. caninum*, *T. leonina*, *U. stenocephala*, *D. immitis*, and *A. vasorum*), as well as against ticks and fleas, is an advantage as it protects dogs against a plethora of endo- and ectoparasites of veterinary and public health importance.

In animals from the control group (G1) that scored positive for eyeworms, the most common clinical signs observed were mild conjunctivitis and ocular discharge, which are among the clinical presentations of ocular thelaziosis by *T. callipaeda*, not only in dogs but also in other animal species [4]. The absence of other symptoms associated with this eyeworm infection in the positive animals evaluated herein could be related to the low parasitic burden (i.e., minimum one and maximum seven adult eyeworms in one or both eyes). Indeed, it is suggested that the number of *T. callipaeda* adults within the eyes is correlated with the severity of the infection [1, 4]. The areas selected for this study provided suitable spots for the realization of the trials, as in these sites, *T. callipaeda* infections in dogs and wild canids such as wolves and foxes have been reported in several studies [9, 19, 22, 24, 33, 34]. Selecting study areas already known to be endemic for this parasite is essential for conducting these kinds of studies aiming to test the efficacy of endo- and ectoparasiticides for the prevention of infections by vector-borne diseases [35]. As *T. callipaeda* is highly endemic in several European countries, the evaluation of products already licensed for other parasites is also an advantage that facilitates their use against thelaziosis in dogs. For example, Simparica Trio is currently licensed in Europe for nematodes, ticks, and fleas [26]. In addition, the same product is licensed for the treatment of parasitic nematode and ectoparasite infestation in dogs in the USA, where the first autochthonous case of *T. callipaeda* in a dog was published in 2021 [36]. This advantage makes the product a good candidate and increases the options for products highly efficacious in treating and preventing infection by this eyeworm in dogs.

The prevention of *T. callipaeda* infecting dogs in highly endemic areas should be considered a priority for public health, as this parasite is well known to infect humans as well, with several reports published worldwide [37–43]. Therefore, since dogs are considered domestic reservoirs and main vertebrate hosts of this nematode [1], preventing the development of infection in these animals could also be a prophylaxis measure for zoonotic infection in humans inhabiting endemic areas [15].

## Conclusion

Data presented herein demonstrate 100% efficacy of Simparica Trio for the prevention of *T. callipaeda* eyeworm infection in dogs from highly endemic areas of France and Italy. Considering that this formulation is currently licensed in Europe (with several countries endemic for *T. callipaeda*) and the USA (first autochthonous case of *T. callipaeda* in a dog detected in 2021) for the treatment of a wide range of endo- and ectoparasites, its use is advantageous for protecting dogs against these parasitic agents, which may indirectly reduce the risk of human infection.

## Supplementary Information

**Additional file1: Table S1.** Severity of the observed clinical signs for the eyeworm-positive dogs (only G1) by visit day.

## Data Availability

The data that support the findings of this study are available from the corresponding author upon reasonable request.

## Abbreviations

GCP: Good clinical practice
G: Group
EU: European Union
DNA: Deoxyribonucleic acid
