# Supplementary material for: Efficacy of a formulation of sarolaner/moxidectin/pyrantel (Simparica Trio®) for the prevention of Thelazia callipaeda canine eyeworm infection
Source: Parasit Vectors. 2022 Oct 16;15:370. doi: 10.1186/s13071-022-05501-6 (PMC9575252; doi:10.1186/s13071-022-05501-6)
Supplement: Supplementary file 1 — Additional file1: Table S1. Severity of the observed clinical signs for the eyeworm-positive dogs (only G1) by visit day. [file 13071_2022_5501_MOESM1_ESM.docx]

Supplementary file

Table S1. Severity of the observed clinical signs for the eyeworm-positive dogs (only G1) by visit day

| Clinical Sign | Eye | Visit Day | Severity | | | | | | Total | |
| --- | --- | --- | --- | --- | --- | --- | --- | --- | --- | --- |
|  |  |  | Absent | | Mild | | Moderate | |  |  |
|  |  |  | *n* | % | *n* | % | *n* | % | *N* | % |
| Conjunctivitis | Left | 0 | 15 | 93.8 | 1 | 6.3 | 0 | 0 | 16 | 100 |
|  |  | 30 | 16 | 100 | 0 | 0 | 0 | 0 | 16 | 100 |
|  |  | 60 | 15 | 93.8 | 1 | 6.3 | 0 | 0 | 16 | 100 |
|  |  | 90 | 10 | 83.3 | 2 | 16.7 | 0 | 0 | 12 | 100 |
|  |  | 120 | 5 | 62.5 | 3 | 37.5 | 0 | 0 | 8 | 100 |
|  |  | 150 | 4 | 100 | 0 | 0 | 0 | 0 | 4 | 100 |
|  |  | 180 | 1 | 100 | 0 | 0 | 0 | 0 | 1 | 100 |
|  | Right | 0 | 15 | 93.8 | 1 | 6.3 | 0 | 0 | 16 | 100 |
|  |  | 30 | 16 | 100 | 0 | 0 | 0 | 0 | 16 | 100 |
|  |  | 60 | 14 | 87.5 | 2 | 12.5 | 0 | 0 | 16 | 100 |
|  |  | 90 | 10 | 83.3 | 2 | 16.7 | 0 | 0 | 12 | 100 |
|  |  | 120 | 6 | 75.0 | 2 | 25.0 | 0 | 0 | 8 | 100 |
|  |  | 150 | 4 | 100 | 0 | 0 | 0 | 0 | 4 | 100 |
|  |  | 180 | 1 | 100 | 0 | 0 | 0 | 0 | 1 | 100 |
|  |  |  |  |  |  |  |  |  |  |  |
| Ocular Discharge | Left | 0 | 15 | 93.8 | 0 | 0 | 1 | 6.3 | 16 | 100 |
|  |  | 30 | 16 | 100 | 0 | 0 | 0 | 0 | 16 | 100 |
|  |  | 60 | 15 | 93.8 | 1 | 6.3 | 0 | 0 | 16 | 100 |
|  |  | 90 | 10 | 83.3 | 2 | 16.7 | 0 | 0 | 12 | 100 |
|  |  | 120 | 6 | 75.0 | 2 | 25.0 | 0 | 0 | 8 | 100 |
|  |  | 150 | 4 | 100 | 0 | 0 | 0 | 0 | 4 | 100 |
|  |  | 180 | 1 | 100 | 0 | 0 | 0 | 0 | 1 | 100 |
|  | Right | 0 | 15 | 93.8 | 0 | 0 | 1 | 6.3 | 16 | 100 |
|  |  | 30 | 16 | 100 | 0 | 0 | 0 | 0 | 16 | 100 |
|  |  | 60 | 14 | 87.5 | 2 | 12.5 | 0 | 0 | 16 | 100 |
|  |  | 90 | 9 | 75.0 | 3 | 25.0 | 0 | 0 | 12 | 100 |
|  |  | 120 | 7 | 87.5 | 1 | 12.5 | 0 | 0 | 8 | 100 |
|  |  | 150 | 4 | 100 | 0 | 0 | 0 | 0 | 4 | 100 |
|  |  | 180 | 1 | 100 | 0 | 0 | 0 | 0 | 1 | 100 |

*n*: number of dogs, %: percentage of all dogs
